# Unanticipated Biatrial 18F-Fluorodeoxyglucose (18F-FDG) Uptake in Diffuse Large B-cell Lymphoma Surveillance PET/CT

**DOI:** 10.7759/cureus.67120

**Published:** 2024-08-18

**Authors:** Pokhraj P Suthar, Sumeet Virmani

**Affiliations:** 1 Department of Diagnostic Radiology and Nuclear Medicine, Rush University Medical Center, Chicago, USA

**Keywords:** clinical, holter-ekg, lymphoma, fdg pet/ct scan, atrial fibrillation

## Abstract

Cardiac lymphomas are rare manifestations of diffuse large B-cell lymphoma (DLBCL), occurring either primarily or secondarily. Imaging modalities such as magnetic resonance/computed tomography (MR/CT) and fluorine-18 fluorodeoxyglucose positron emission tomography/computed tomography (FDG PET/CT) are pivotal for accurate detection and staging. However, cardiac FDG uptake during oncological PET/CT scans lacks specificity, necessitating further investigation into abnormal patterns, particularly in atrial FDG uptake, which may indicate underlying cardiac arrhythmias. A significant proportion of atrial fibrillation (AF) patients exhibit heightened FDG uptake in the atrium, correlating with an increased stroke risk. We present an asymptomatic 81-year-old female with DLBCL, hypertension, and AF, incidentally found to have abnormal biatrial FDG uptake on surveillance PET/CT. This case underscores the importance of comprehensive cardiology evaluation in asymptomatic oncology patients to manage potential cardiac complications effectively. Advanced imaging techniques and integrated care strategies are crucial for optimizing outcomes in cardiac lymphoma patients.

## Introduction

Cardiac lymphomas are exceedingly rare, and the involvement of the myocardium in diffuse large B-cell lymphoma (DLBCL) can manifest as either primary or secondary. Magnetic resonance/computed tomography (MR/CT) imaging is preferred for a more accurate depiction of cardiac lymphomas [1]. While performing oncological 18F-fluorodeoxyglucose (18F-FDG) PET/CT, it is essential to note that cardiac FDG uptake is nonspecific, and an abnormal atrial FDG uptake pattern may necessitate further investigation to rule out any underlying cardiac arrhythmia [2, 3]. Patients with hypertension have an elevated risk of developing cardiac arrhythmias [4]. Furthermore, the study conducted by Sinigaglia et al. highlighted that approximately one-third of patients with atrial fibrillation (AF) exhibited diffuse, increased FDG uptake in the atrium, which consequently increases their risk of stroke [5]. This case underscores the importance of considering cardiac arrhythmia as a potential cause of abnormal atrial FDG uptake in asymptomatic patients undergoing oncological PET/CT imaging. The incidental finding of abnormal FDG uptake in both atria of a patient with cutaneous follicular-type DLBCL, despite no symptoms, led to the detection of AF and resolution of the uptake after a year with Holter monitoring. The rarity of cardiac lymphomas highlights the need for MR/CT imaging for accurate diagnosis. Given the nonspecific nature of cardiac FDG uptake, especially in hypertensive patients, a thorough cardiology evaluation is vital for managing potential arrhythmias in oncology settings.

## Case presentation

We present the case of an 81-year-old asymptomatic female with a history of cutaneous follicular-type DLBCL, undergoing surveillance 18F-FDG PET/CT. The patient had experienced multiple recurrences and was managed with rituximab and the DeAngelis protocol. Her medical history included hypertension, obesity, and AF, treated with metoprolol (75 mg per day) and therapeutic anticoagulation using warfarin (2.5 mg per oral daily). She denied chest pain, shortness of breath, weight loss, recent travel, or trauma. A review of systems revealed no fevers, chills, or night sweats, with good energy levels, stamina, and no limitations in daily activities. She had a healthy appetite without recent weight loss or early satiety. There were no headaches, dizziness, visual changes, cough, or respiratory distress. A cardiac review showed no chest pain, and gastrointestinal and genitourinary systems were normal without diarrhea, constipation, melena, or dysuria. Musculoskeletal review was unremarkable with no new pain, and there were no neurological deficits or psychiatric symptoms. Hematology and lymphatics review showed no enlarged lymph nodes or increased bruising.

Clinical examination revealed a blood pressure of 167/76 mmHg, heart rate of 70 beats per minute, temperature of 97 °F (36.1 °C), respiratory rate of 16 breaths per minute, height of 147.5 cm, weight of 60.3 kg (133 lb), SpO2 of 99%, and body mass index of 27.73 kg/m². Laboratory analysis indicated hyperproteinemia and anemia (Table 1). A prior skin biopsy confirmed recurrent B-cell lymphoma, follicular type, with immunohistochemical staining showing predominantly CD20-positive B-cells with admixed CD3-positive T-cells. B-cells were positive for BCL-2 and variably positive for BCL-6, with a moderate proliferative index (Ki-67 ~50%).

**Table 1:** Laboratory work-up WBC: white blood count, RBC: red blood count, BUN: blood urea nitrogen, ALP: alkaline phosphatase, AST (SGOT): aspartate aminotransferase (serum glutamic-oxaloacetic transaminase), ALT (SGPT): alanine aminotransferase (serum glutamic-pyruvic transaminase), LDH: lactate dehydrogenase, PT-INR: prothrombin time - international normalized ratio, aPTT: activated partial thromboplastin time.

Laboratory test	Laboratory value	Reference range
White blood count	5.68 k/µL	4.00–10.00 k/µL
Red blood count	3.75 M/ µL	4.40–6.40 M/ µL
Hemoglobin	11.7 g/dL	12.0–18.0 g/dL
Platelet count	8.6 k/µL	150–450 k/µL
Sodium	138 mmol/L	137–147 mmol/L
Potassium	4.4 mmol/L	3.4–5.3 mmol/L
Chloride	99 mmol/ L	99–108 mmol/ L
CO_2_ total	28 mmol/L	22–29 mmol/L
Urea nitrogen (BUN)	14 mg/dL	8–21 mg/dL
Creatinine	0.35 mg/dL	0.75–1.20 mg/dL
Glucose	113 mg/dL	60–99 mg/dL
Calcium	8.8 mg/dL	8.7–10.7 mg/dL
Total bilirubin	0.36 mg/dL	0–1.2 mg/dL
Alkaline phosphatase	111 U/L	55–142 U/L
AST (SGOT)	16 U/L	0–32 U/L
ALT (SGPT)	21 U/L	0–33 U/L
LDH	158 U/L	135–214 U/L
Total protein	6.2 g/dL	6.4–8.3 g/dL
Albumin	4.4 g/dL	3.5–5.2 g/dL
PT-INR	1.0	0.83–1.23
aPTT	23.6 seconds	23–33 seconds

**Figure 1:**
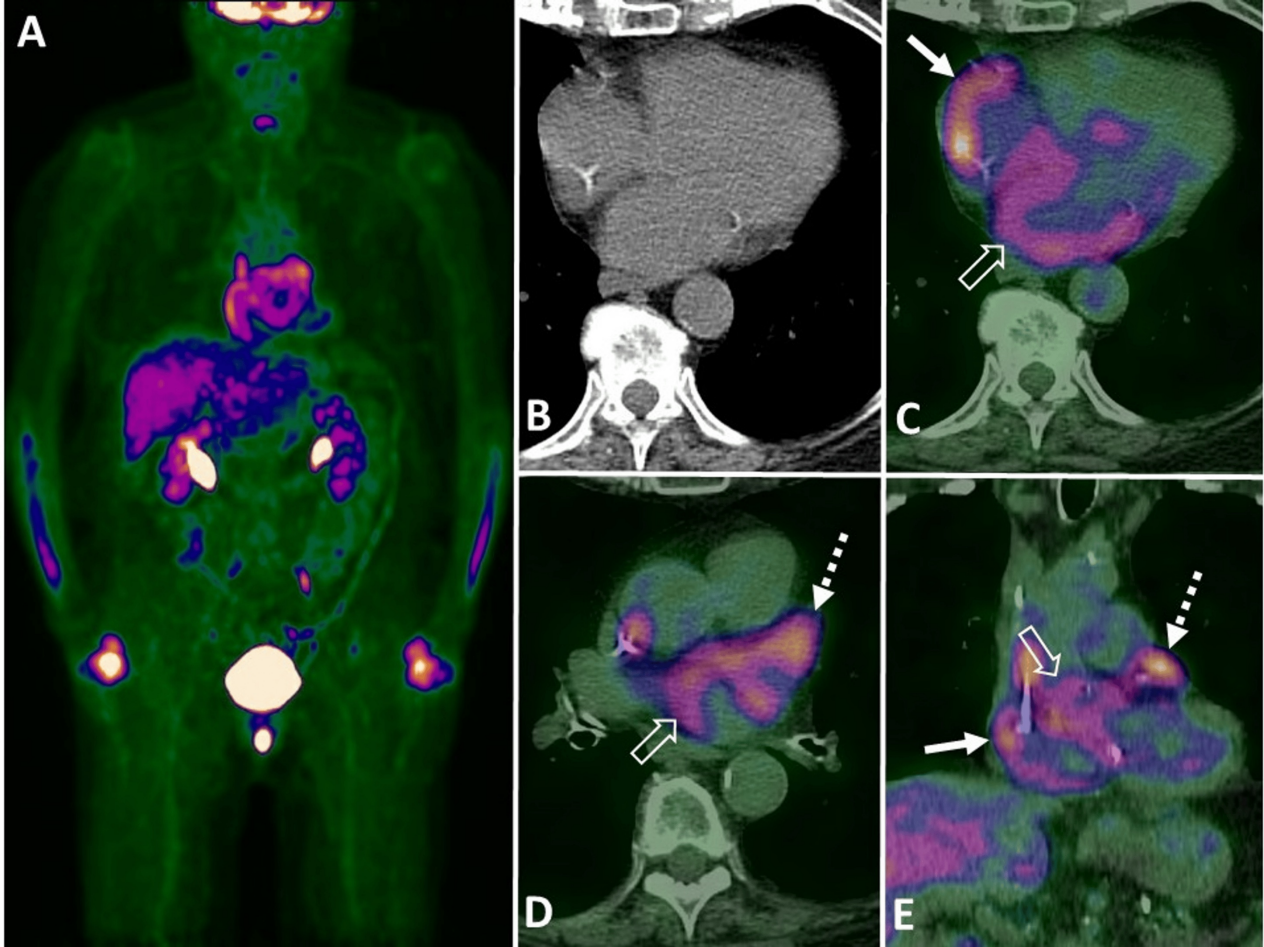
Surveillance fluorine 18 (18F) fluorodeoxyglucose (FDG) PET/CT images (A) maximum intensity projection, (B) unenhanced axial CT image, (C-D) transaxial fused PET/CT images, and (E) coronal reformatted fused PET/CT image demonstrates abnormal focus of increased FDG uptake in the right atrium (solid white arrow in C and E), left atrium (open white arrow in C-D) and left atrial appendage (dashed white arrow in D-E) without structural abnormality in unenhanced CT.

**Figure 2:**
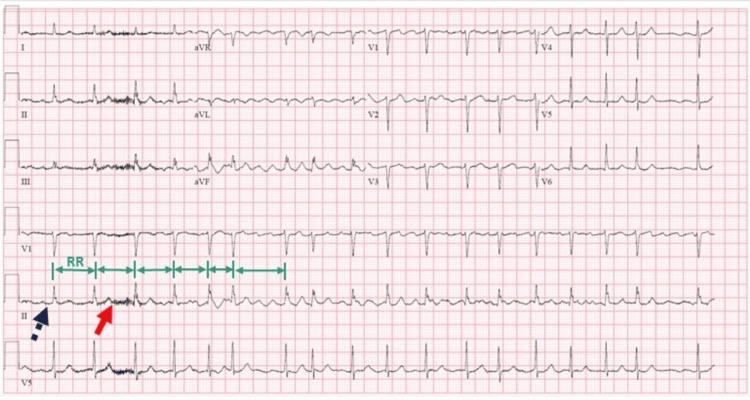
An electrocardiogram shows irregular RR interval, fibrillatory waves (red arrow), and no distinguishable P waves (purple dashed arrow) suggestive of features of atrial fibrillation.

**Figure 3:**
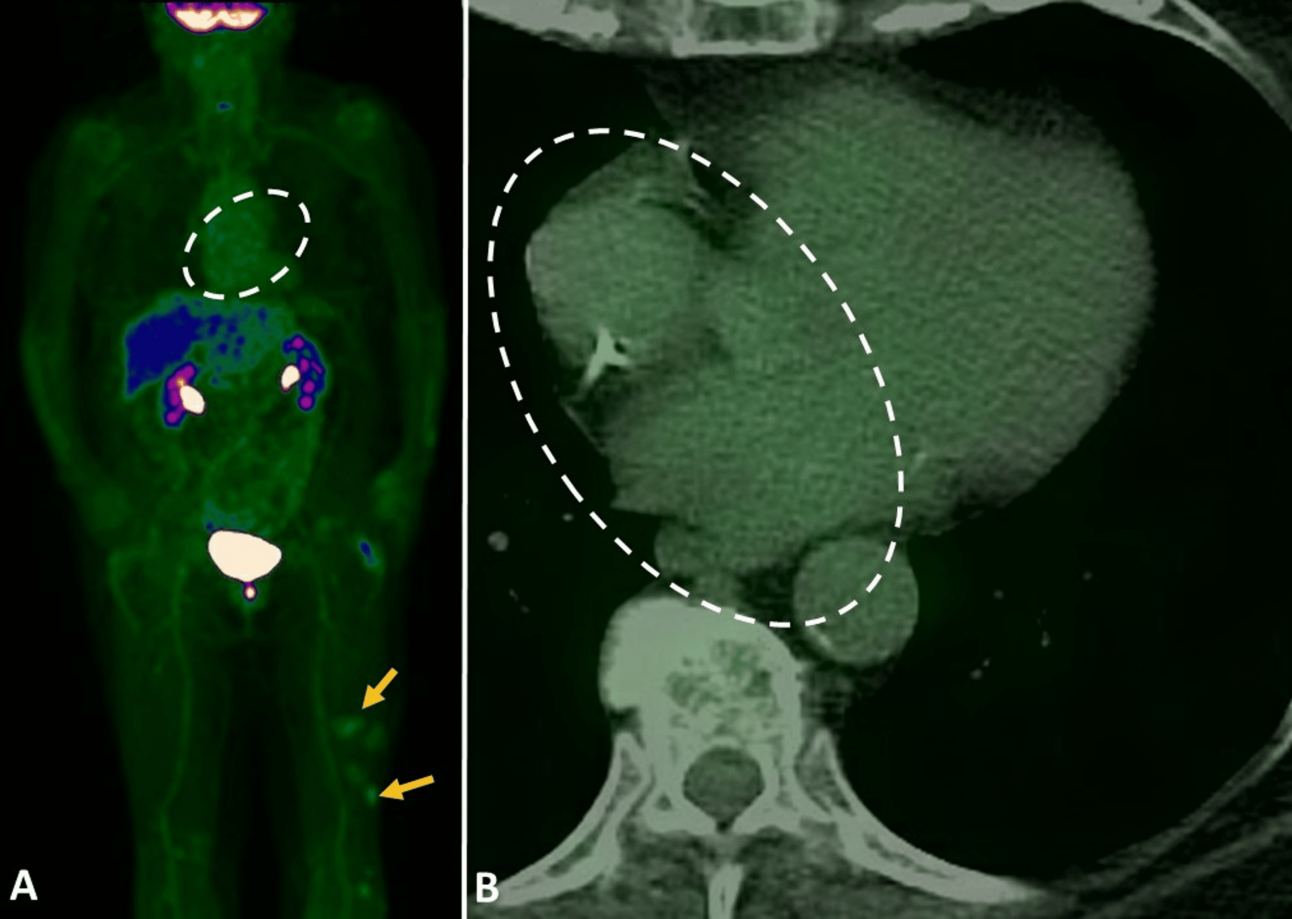
One-year follow-up 18F-FDG PET/CT images (A) maximum intensity projection and (B) transaxial fused PET/CT images demonstrate resolution of biatrial FDG uptake (dashed open oval circle in A and B). Mild foci of FDG uptake in the left thigh (yellow arrows in A) from cutaneous follicular-type diffuse large B-cell lymphoma (DLBCL).

Imaging with surveillance 18F-FDG PET/CT incidentally revealed abnormal FDG uptake in both atria without structural abnormalities on CT. No abnormal uptake was noted elsewhere. Deauville PET criteria indicated a score of 1 (Figure 1). An electrocardiogram showed an irregular RR interval, fibrillatory waves, and no distinguishable P waves suggestive of AF (Figure 2). One-year follow-up 18F-FDG PET/CT demonstrated resolution of abnormal biatrial FDG uptake (Figure 3).

## Discussion

Lymphoma typically affects the heart secondarily due to widespread disease, as noted in autopsy studies where it is found in 8.7%-25% of patients, constituting 9% of all metastases to the heart [6-8]. Cardiac lymphomas are rare, with myocardial involvement in DLBCL being either primary or secondary. Primary cardiac lymphoma (PCL) is sporadic, comprising less than 0.5% of extranodal lymphomas and less than 2% of resected cardiac tumors [8]. Most cardiac lymphomas are of B-cell origin, with DLBCL being the most common. PCL usually affects the right heart, while disseminated lymphoma typically involves the epicardium (61%) and myocardium, with the left ventricle and right atrium affected in 55% and 54% of cases, respectively [8].

Cardiac involvement in secondary lymphoma often goes unnoticed until death due to its frequently asymptomatic or nonspecific symptoms. When present, symptoms can include heart failure, chest pain, superior vena cava (SVC) syndrome, arrhythmia, embolic phenomena, and hemodynamic instability, but ECG and chest X-rays are generally not sensitive or specific for detection [9, 10]. According to the nationwide study by Fawzy et al. of hospitalized patients, AF is associated with an increased risk of cardiac arrest [11].

According to Yun et al., all hematologic malignancies, such as lymphoma (adjusted subdistribution hazard ratio (HR): 2.29; 95% CI: 2.10-2.51), leukemia (HR: 2.64; 95% CI: 2.38-2.92), and multiple myeloma, carry a high risk of developing AF, as do intrathoracic cancers among solid tumors, including lung (HR: 2.39; 95% CI: 2.30-2.48), esophageal (HR: 2.69; 95% CI: 2.45-2.95), and CNS cancers (HR: 2.62; 95% CI: 2.35-2.91) [12]. Several factors contribute to the high risk of AF observed in patients with cancer. Firstly, cancer and AF share common risk factors, with age being the most significant risk factor for both conditions [13]. However, patients with a history of cancer had a higher risk of AF even after adjusting for common risk factors, suggesting another mechanism may be responsible. Additionally, many cancer therapies, including surgery and systemic treatments, are associated with new-onset AF [14]. Patients with AF can be asymptomatic or present with symptoms like palpitations, (near) syncope, chest pain, or fatigue. AF is categorized as paroxysmal, (long-standing) persistent, or permanent based on clinical presentation and episode duration (>30 seconds documented by ECG or similar monitoring). Mobile health devices such as smartphones and wearable patches aid early AF detection, but false positives necessitate physician review. Traditional ECGs cannot assess AF persistence or severity of atrial electropathology, underscoring the need for advanced diagnostic tools to personalize AF diagnosis and treatment [15].

AF therapy aims to eliminate AF episodes, restore sinus rhythm, normalize atrioventricular synchrony, and enhance the atrial contribution to stroke volume. The 2020 ESC guidelines recommend a structured approach involving confirmation of AF, characterization of patients using the 4S-AF scheme (CHA2DS2VASc score, EHRA symptom score, AF burden, and substrate severity), and implementation of the Atrial fibrillation Better Care (ABC) pathway [15]. This comprehensive strategy includes "A" (Avoid stroke/Anticoagulation), "B" (Better symptom management with patient-centered decisions on rate or rhythm control), and "C" (Cardiovascular risk and comorbidity optimization, addressing lifestyle changes and patient preferences).

Primary cutaneous diffuse large B-cell lymphoma often appears to affect only a small skin area, but the disease is frequently more extensive than it seems. Treatment usually involves rituximab combined with systemic chemotherapy, typically R-CHOP (rituximab, cyclophosphamide, doxorubicin, vincristine, and prednisone), though other regimens may also be considered. If there is central nervous system (CNS) involvement, the DeAngelis protocol is an appropriate treatment option [16].

MR/CT imaging is preferred for detailed visualization of cardiac lymphomas [1]. 18F-FDG PET/CT is employed for the initial staging and surveillance of lymphoma, detecting increased FDG uptake in lymph nodes and organ infiltrations, which aids in determining the Deauville PET score [17]. During oncological 18F-FDG PET/CT scans, it is crucial to recognize that cardiac FDG uptake lacks specificity, and abnormal patterns in atrial FDG uptake may require further investigation for potential underlying cardiac arrhythmias [2, 3]. Sinigaglia et al. found that about one-third of atrial fibrillation patients exhibit diffuse, increased FDG uptake in the atrium, heightening their stroke risk [5]. Our case emphasizes the importance of considering cardiac arrhythmias as a possible cause of abnormal atrial FDG uptake in asymptomatic patients undergoing oncological PET/CT imaging, underscoring the need for comprehensive cardiology evaluation to mitigate potential complications.

## Conclusions

In conclusion, this case underscores the importance of thorough diagnostic evaluation when encountering abnormal FDG uptake in asymptomatic patients during oncological PET/CT scans. The incidental finding of atrial FDG uptake in a patient with cutaneous follicular-type DLBCL, despite the absence of symptoms, led to the diagnosis of atrial fibrillation and subsequent resolution of uptake with Holter monitoring. The rarity of cardiac lymphomas emphasizes the need for MR/CT imaging to ensure accurate diagnosis. Given the nonspecific nature of cardiac FDG uptake, particularly in hypertensive individuals, a comprehensive cardiology evaluation is essential for the proactive management of potential arrhythmias in oncology settings. This highlights the necessity of detecting abnormal FDG uptake and the need for a more specifically described comprehensive diagnostic strategy for better understanding.
